# The Accuracy of the I2 Statistic for Detecting Baseline Imbalances and Selection Bias in Randomized Controlled Trials: A Simulation Study

**DOI:** 10.7759/cureus.112766

**Published:** 2026-07-16

**Authors:** Steffen Mickenautsch, Veerasamy Yengopal

**Affiliations:** 1 Faculty of Dentistry, University of the Western Cape, Cape Town, ZAF; 2 Community Dentistry, University of the Witwatersrand, Johannesburg, ZAF

**Keywords:** bias test, i2 test, review of clinical trials, selection bias, systematic review and meta analysis

## Abstract

Aim: To assess the accuracy of the I^2^ statistic, applied as a trial-adjusted, simulated comparator trials (SCTs) based I^2^ test for detecting baseline imbalances and selection bias in randomized controlled trials (RCTs).

Methods: Fifty non-biased and 50 biased trials set at 40% selection bias severity were simulated in MS Excel (Microsoft Corporation, Redmond, Washington, USA). All trials were tested using statistical significance testing and the I^2^ test. Null hypotheses were tested that the sensitivities and specificities of both tests did not statistically significantly differ and that the selection bias category scores, established by the I^2^ test, do not statistically significantly correlate with the p-values from statistical significance testing. McNemar test and Spearman's rank correlation were used. The risk of the I^2^ test overestimating selection bias severity was also examined.

Results: I^2^ test demonstrated higher sensitivity than significance testing for detecting both baseline imbalance and selection bias (p < 0.0001). Specificity for baseline imbalance detection was identical at 100% for both methods; 95% CI: 90%-100%. For selection bias, specificity was greater with significance testing than with the I² test (p = 0.0005). A significant negative, large (0.5 ≤ |r|) correlation emerged between I² scores and significance test p-values: Spearman’s r = -0.61, p < 0.0001. All null hypotheses, with the exception of the test specificities for baseline imbalance detection, were rejected. The I^2^ test caused 26% of ‘true positive’ trials to be over-classified by one severity category.

Conclusion: The I^2^ test was significantly more accurate for identifying baseline imbalance and selection bias detection, and equally accurate for ruling out baseline imbalance compared with statistical significance testing. However, the I^2^ test was significantly less accurate in detecting selection bias absence. The I^2^ test's bias category scores were largely negatively associated with p-values and tended to overestimate bias severity. Negative I^2^ test results appear reliable for ruling out baseline imbalances and selection bias in RCTs, while positive I^2^ test results are highly accurate to detect baseline imbalances but should be interpreted cautiously when assessing selection bias.

## Introduction

Systematic reviews of randomized controlled trials (RCTs) are essential tools that allow for a more objective appraisal of the current clinical evidence and the identification of areas where further studies are required [1]. A key component of such reviews is assessing the risk of selection bias. Selection bias in RCTs is generated by either the non-random, erroneous, or willful allocation of patients with known treatment-success-supporting characteristics to one of the trial treatment groups, which may lead to an artificially more favorable outcome in one group than the other [2]. Such a type of allocation generates imbalances in baseline characteristics between treatment groups. Consequently, appraisal of selection bias risk includes the assessment of whether baseline characteristics differ systematically across RCT intervention groups. These characteristics, such as patients' age, height, and medical conditions, are collected prior to randomization and summarized as mean values with standard deviation (SD) or median values with interquartile range (IQR) per treatment group during RCTs [3].

Corbett et al. (2014) proposed to include the assessment of baseline imbalances between treatment groups as a routine part of systematic reviews to reduce uncertainty in systematic review conclusions and reduce the risk of chance findings being ascribed to treatment effects [4]. Since it is not possible during a systematic review to know ahead of time whether there is no selection bias present in an RCT, a strong case was made for formal hypothesis testing of baseline variables to identify statistically significant (p < 0.05) baseline imbalances [5,6].

However, statistical significance testing has recognized limitations [6]. It cannot differentiate chance variation from selection bias as a reason for statistically significant baseline imbalances [7]. The Cochrane Collaboration makes a clear distinction between both, stating that chance imbalances are not a source of systematic error [8]. Nevertheless, chance imbalances in baseline characteristics can also affect the trial effect estimate between trial intervention groups [9,10]. Given this, any deviation from the true treatment effect caused by imbalances in baseline characteristics (whether caused by selection bias or chance--also sometimes referred to as ‘chance bias’ [9]) appears to be important. This supports the routine testing for baseline imbalances during critical trial appraisal during systematic reviews.

However, statistical significance testing may not be the best choice for such a purpose. A simulation study investigating test accuracy for identifying selection bias that specifically excluded chance effect established a low sensitivity for statistical significance testing (24%; 95%CI: 13%-38%) that statistically significantly (p < 0.0001) differed from the sensitivity of the trial-adjusted, SCT-based I^2^ test: 76% (95%CI: 62%-87%) [11]. This test was developed for detecting selection bias in single RCTs with the rationale that effective randomization in RCTs assures a lack of in-between study heterogeneity beyond the play of chance, which is reflected as I^2^ = 0% in a baseline variable meta-analysis [12]. Despite these promising results, it remains unclear whether the I^2^ test may generally identify baseline imbalance and selection bias more accurately than statistical significance testing when chance variation is present. It is also uncertain how the selection bias category scores, established by the I^2^ test for any RCT, are related to the p-values from statistical significance testing. A further question is whether the significantly higher sensitivity of the I^2^ test [11] is connected with any tendency to overestimate actual selection bias severity. Answers to these questions will determine how useful the I^2^ test might be as a possible tool for the critical RCT appraisal during systematic reviews.

Against this background, this simulation study aimed to evaluate the accuracy of the trial-adjusted, SCT-based I^2^ test for detecting imbalances of baseline variables and selection bias in RCTs. Its primary objectives were to establish the I^2^ statistic's test sensitivity and specificity for identifying baseline imbalances in general and selection bias in particular, in comparison to that of statistical significance testing. The null hypotheses were tested that the sensitivities and specificities of both methods do not statistically significantly differ and that the selection bias category scores, established by the I^2^ test, do not statistically significantly correlate with the p-values established by statistical significance testing. A secondary objective was to investigate whether classification by I^2^ test bias categories leads to overestimation of actual selection bias severity in RCTs.

This article was previously posted as a preprint in Research Gate on June 26, 2026 [13].

## Materials and methods

Generation of simulation trials

Trials were simulated in MS Excel (Microsoft Corporation, Redmond, Washington, USA), each containing two treatment groups, A and B. Following a previous simulation model [11], each simulation trial consisted of three parallel data columns: Column 1: a sequence of subject ID (accession) numbers representing trial subjects; Column 2: a random sequence of allocation to treatment Group A or B and Column 3: a sequence of values of a simulated baseline variable that were drawn randomly from a normal distribution around the median height. The random allocation sequences for column 2 were generated by block-randomization with block size 4 using the ‘Sealed Envelope’ online tool [14] for all simulated trials and are presented in the Appendix (Section 1). The sequences of simulated baseline values in column 3 were generated using an online random number generator [15]. The comprehensive version of the generator was used for randomly selecting the values for each subject with the following settings: Allow duplication of results? = Yes; Type of result to generate = Decimal; Precision = 2 digits. To introduce chance values, they were retained in their original random order without sorting.

To generate realistic baseline values, trials were randomly selected from 332 clinical RCTs that were included in a previous study [16]. From these trials, the values of the baseline variable ‘age’ from two treatment groups and the total trial sample sizes were extracted. The mean baseline values with standard deviation (SD) from both groups were combined, and the minimum/maximum values per trial at one standard deviation range (Sample mean ± SD) were calculated. Any reported standard error (SE) was converted to SD using the formula: $SD = SE\sqrt{N}$ Median values with minimum/maximum range or interquartile range (IQR) were converted to mean (SD) values using the formulas by Hozo et al. (2005) [17] and Wan et al. (2014) [18], respectively. Details of the random trial selection process, selected RCTs, and extracted trial data are presented in the Appendix (Section 2). The calculated baseline minimum/maximum values and the extracted RCT sample sizes were entered into the online random number generator [15] to generate baseline values for column 3 and the sample sizes for the simulated trials.

Half of the simulated trials were left non-biased, while the other half were biased by assigning 40% of subjects preferentially to one group. Selection bias was introduced by first sorting the random values in column 3 into ascending order. In the next step, the random allocation sequence in column 2 for the first 40% of the trial subjects was sorted in alphabetical order, thus ensuring that lower baseline values from column 3 were artificially assigned to group A and higher ones to Group B. Data for all non-biased and biased simulated trials are presented in the Appendix (Section 3). 

Sample size calculation

The sample size was calculated using the formula by Buderer et al. (1996) [19]. The following parameters were used: expected sensitivity and specificity = 85%; prevalence of disease (i.e., prevalence of biased trials) = 50%; expected precision = 10%; confidence level 100 (1-a) = 95%; expected dropout rate = 0%. Accordingly, the calculation generated a required sample size of 98 trials, which was rounded up to 100. Of these, 50 simulated trials were assigned selection bias, and 50 remained unbiased.

Statistical significance testing

For each simulation trial, the mean values (SD) of the baseline variable in column 3 were calculated separately for Groups A and B and together with each group’s sample size (N) statistically compared. Review Manager (RevMan) 5.0.24 software [20] was used for the analyses. The mean difference (MD) with 95% confidence interval (CI) and p-value were recorded per trial. A result was considered positive if p < 0.05, and negative if p > 0.05. Results with p-values = 0.05, with the lower confidence limit below and the upper confidence limit at or above zero values, were also considered as negative test results.

I^2^ test

The trial-adjusted, SCT-based I^2^ test with revised I^2^ threshold limits for five bias categories was used [12,16]. A step-wise description of the test together with its five bias categories is presented in the Appendix (Section 4). For each simulation trial, two simulated comparator trials (SCTs) were generated in MS Excel using the three data columns. Mean values with SD for groups A and B from each SCT were entered into a fixed-effect meta-analysis for continuous data. For each SCT, the random sequence of baseline values in Column 3 was sorted in ascending order before sorting according to the random allocation sequence in Column 2. Data from the simulated RCT were added into the meta-analysis, and the I^2^ point estimate was computed for each of the sample sizes (n_i_) = 98, 99, and 100 set per group for all three trials, using Cochrane's Review Manager (RevMan) software [20]. The bias category was assigned based on the three resulting I² point estimates. Category 1 was classified as a negative result, indicating absence of imbalance/bias, while categories 2-5 were classified as positive, indicating the presence of baseline imbalances and selection bias [12].

Test accuracy measurement

The numbers of true positive (TP), false positive (FP), false negative (FN), and true negative (TN) test results were established for statistical significance testing and the I^2^ test. Because, I^2^ point estimates above 0% (corresponding to selection bias category 1) are mathematically not possible without any baseline imbalance, i.e., any difference between baseline variable mean (SD) values of two trial treatment groups [21], all identified baseline imbalances equivalent to the bias categories 2-5 [12] were considered as ‘true positive’ I^2^ test results (TP). Because, the same set of data was tested using both tests, any statistical significance test result with p < 0.05 for trials with I^2^ = 0% was considered ‘false positive’ (FP).

From the TP, FP, TN, and FN values, sensitivity and specificity with 95% CI (in %) were calculated for both tests. The test sensitivity was defined as the probability of obtaining a positive result when baseline imbalances or selection bias were present: TP/(TP + FN). The test specificity was defined as the probability of a negative result when baseline imbalances or selection bias were absent (calculated as TN/(FP + TN)) [22]. 

Null-hypotheses testing

The null hypotheses for baseline imbalances and selection bias were tested by statistically comparing the established sensitivities and specificities between statistical significance testing and the I^2^ test using McNemar’s test. The null hypotheses were tested that the sensitivities and specificities of both tests did not significantly differ. Alpha was set at 5%.

The null hypothesis for the association between the selection bias category scores and p-values was tested by Spearman’s rank correlation coefficient. P-values from statistical significance testing served as the independent (x) and the selection bias category scores, established by the I^2^ test [12], the dependent variable (y) for analysis. The alpha level was set at 5%. The analyses were carried out in SAS (SAS Institute, Cary, North Carolina, USA).

To determine whether the I^2^ test's selection bias category scores overestimated true selection bias severity, the percentage of test results assigned to categories 3-5 instead of category 2 was calculated and expressed as a percentage of all TP results.

## Results

Detailed TN, FN, TP, and FP data are presented in Table 1, and all established p-values and selection bias categories per simulation trial for both tests are presented in the Appendix (Section 5).

**Table 1:** Test results. FP: false positive; TN: true negative; FN: false negative; TP: true positive.

Testing for		Selection bias		Baseline imbalance	
Type of test		Statistical significance testing	I^2^ test	Statistical significance testing	I^2^ test
Type of test results	FP	1	13	0	0
	TN	49	37	37	37
	FN	34	8	46	8
	TP	16	42	17	55

The established sensitivities and specificities of both tests are presented in Table 2. The sensitivity for statistical significance testing and the I^2^ test concerning baseline imbalance detection were 27% (95% CI: 17%-40%) and 87% (95% CI: 76%-94%), respectively. McNemar's test revealed a statistically significant difference between test sensitivities, in favor of the I² test (Table 3; p < 0.0001). Neither test produced false positives, so specificity for baseline imbalance absence was 100% (95% CI: 90%-100%) for both.

**Table 2:** Test accuracy. PE: point estimate in %; CI: confidence interval in %.

Type of test		Statistical significance testing		I^2^ test	
Test sensitivity		PE	95% CI	PE	95% CI
Testing for	Selection bias	32	20-47	84	71-93
	Baseline imbalance	27	17-40	87	76-94
Test specificity		PE	95% CI	PE	95% CI
Testing for	Selection bias	98	89-100	74	60-85
	Baseline imbalance	100	90-100	100	90-100

The sensitivity for statistical significance testing and the I^2^ test concerning selection bias detection were 32% (95% CI: 20%-47%) and 84% (95% CI: 71%-93%), respectively. McNemar's test revealed a statistically significant difference between test sensitivities, in favor of the I² test (Table 3; p < 0.0001). The specificity for statistical significance testing and the I^2^ test concerning selection bias were 98% (95% CI: 89%-100%) and 74% (95% CI: 60%-85%), respectively. McNemar's test revealed a statistically significant difference between test sensitivities, in favor of statistical significance testing (Table 3; p = 0.0005).

**Table 3:** Comparison of test accuracy. Statistically significant difference: *In favor of I^2^ test; **In favor of Statistical significance testing.

I^2^ test	Baseline imbalance				Selection bias			
	Statistical significance testing							
	Test sensitivity		Test specificity		Test sensitivity		Test specificity	
	Negative	Positive	Negative	Positive	Negative	Positive	Negative	Positive
Negative	8	0	37	0.1	8	0	37	0
Positive	38	17	0.1	0	26	16	12	1
McNemar test statistic	38		0		26		12	
p-value	<0.0001*		1.00		<0.0001*		0.0005**	

There was a significant, negative, large (0.5 ≤ |r|) correlation between the p-values from statistically significant differences and the I^2^ test's selection bias category scores: (Spearman's) r = -0.61, p < 0.0001 (see Appendix, Section 6). All null hypotheses were rejected except for the comparison of specificity for baseline imbalance detection.

Of 42 TP results from the 50 biased trials analyzed with the I² test, 11 were assigned category 3 instead of category 2. This suggests that 26% of true-positive trials are being over-classified by one severity category (see Appendix, Section 5).

## Discussion

The objectives of this study were to test the null hypotheses that the sensitivity and specificity of the I² test for detecting baseline imbalance and selection bias do not differ significantly from those of statistical significance testing, that I² test selection bias category scores do not correlate significantly with p-values from statistical significance testing, and that I² test selection bias categories do not overestimate the true selection bias severity in RCTs.

The study results showed that the I^2^ test was significantly more accurate in identifying baseline imbalances and selection bias, but equally accurate in identifying lack of baseline imbalances, as shown by statistical significance testing in the simulation studies. The test was significantly less accurate in identifying the absence of selection bias. The I^2^ test's generated point estimates were largely negatively associated with the p-values generated by statistical significance testing and its established selection bias categories caused 26% of ‘true positive’ trials being over-classified by one severity category. In contrast to a previous investigation on this topic [11], the current study specifically included chance into its stimulation model, added baseline imbalance in addition to selection bias detection and applied revised I^2^ threshold limits for five bias categories [12]. In addition, category-score-to-p-value correlation and the severity-overestimation analysis were included.

The current findings partly agree with previous study findings, in terms of higher test sensitivity of the I^2^ test compared to statistical significance testing for selection bias detection [11]. However, while past study results showed equivocal specificity for both tests (100%; 95% CI: 93%-100% [11]), the current study establishes significantly lower specificity for the I^2^ test (74%; 95% CI: 60%-85% compared to 98%; 95% CI: 89%-100% for statistical significance testing). This discrepancy is unlikely to be explained by threshold differences. In the previous investigation, the baseline variable meta-analysis was conducted with varying sample sizes per SCT group (n_i_ = 100, 200, 400, and 600) [11], while the current study was conducted with the sample sizes for all trial groups (n_i_ = 98, 99, and 100). A lower n_i_ reduces the I^2^ point estimate in a baseline variable meta-analysis and thus increases the I^2^ test’s cut-off point [21]. A higher cut-off point would be expected to increase, not decrease, specificity [23].

Another possible explanation may relate to previous findings that the test sensitivity increased for both tests with increasing selection bias severity [11]. In the present study, all simulation trials were subjected to a higher bias severity (40% of subjects that were non-randomly allocated in favor of one group above the other), while in the previous investigation, a step-wise ascending bias severity from 32% to 40% was used [11], thus resulting in higher sensitivities for both tests in the current study. Since an inverse relationship between test sensitivity and specificity exists [23], the higher test sensitivities in the current study (I^2^ test = 84% versus 76% on the previous study [11]; Statistical significance testing = 32% versus 24%) may have resulted in the lower test specificities (I^2^ test = 74% versus 100%; Statistical significance testing = 98% versus 100%).

Based on these results, the I^2^ test's high sensitivity suggests it may help rule out both baseline imbalances and selection bias in RCTs when the results are negative. However, specificity for selection bias detection was below 80%, and the test tended to overestimate true bias severity. Therefore, positive I^2^ test results need to be considered with caution when appraising specifically for selection bias. Furthermore, the I^2^ test for selection bias detection generated 13 false positive results, indicating it cannot reliably distinguish between baseline imbalances caused by chance from that caused by the actual selection bias effect.

The large, negative association between I^2^ test’s bias categories and p-values from statistical significance testing suggests that p-values could help guide selection of baseline variables for I^2^ testing. Specifically, the baseline variable with differences yielding the lowest p-value (even if above the 5% threshold) may be indicative for the actual highest selection bias category score relevant for a particular trial and thus may need to be tested first.

Possible application of the I^2^ test during systematic reviews of randomized controlled trials (RCTs)

When used in critical appraisal of RCTs during systematic reviews, a negative test result of a baseline covariate with the lowest p-value may corroborate the assumption that any meaningful baseline imbalances and, subsequently, any selection bias effect is absent in the appraised trial. Any positive test result may be judged in conjunction with additional assessment, whether the tested baseline variable may have a strong association with the trial outcome or not. If such an association exists, further trial appraisal may ascertain whether the trial authors included any statistical adjustment, e.g., by using analysis of covariance [24] in regard to the particular baseline imbalance in their data analysis. If an adjustment was performed, the effect of the baseline imbalance may have been corrected for and may not have negatively affected the trial outcome. Because the inclusion of stratification or minimization in the randomization process may prevent ‘chance bias’ [9], any baseline imbalance identified using the I^2^ test may be considered an indication for the presence of selection bias when the inclusion of stratification, e.g., by rank ordering trial subjects according to the baseline variable scores, or minimization was reported in the trial’s method section.

Due to the apparent inability of the I^2^ test to distinguish between chance baseline imbalances and imbalances caused by selection bias, the main utility during RCT appraisal may be the detection of the baseline imbalance, regardless of cause, rather than specifically detecting selection bias in RCTs.

Statistical testing for baseline imbalance has been extensively criticized in the past [9,25-27]. Such critique was directed against testing as part of the RCT methodology by trial authors themselves. In contrast, independent reviewers conducting a systematic review of RCTs cannot know beforehand that no selection bias exists in the reviewed trials or whether chance baseline imbalances may have affected trial outcomes [5,6]. Therefore, baseline imbalance testing maybe a useful adjunct measure during the recommended critical evaluation whether baseline characteristics of trial treatment groups are systematically different or not [4]. Based on the current and previous study results [11], compared to common statistical significance testing, the current version of the trial-adjusted, SCT-based I^2^ test [12] may be a more reliable tool for this purpose than standard statistical significance testing, due to its high sensitivity despite limited specificity and tendency to overestimate bias severity.

Study limitations

Like all trial simulation studies, this study is limited by its reliance on idealized model assumptions that rarely reflect real-world conditions. This disparity may therefore have affected the generalizability of this study’s findings. To address this, we used baseline variables from actual patients and sample sizes that were characteristic of actual clinical trials (see Appendix, Section 2).

Furthermore, all biased simulation trials used a single fixed selection bias level at 40% non-random allocation in favor of one group. In real-world RCTs, such a bias level would vary between trials. In a previous simulation study, it has been observed that the sensitivity of both the I^2^ test and statistical significance testing increases with increasing selection bias level [11]. Therefore, actual test sensitivities would be expected to vary with higher or lower bias severity.

## Conclusions

The trial-adjusted, SCT-based I^2^ test was significantly more accurate in identifying baseline imbalances and selection bias, but equally accurate in identifying lack of baseline covariate imbalances, than statistical significance testing in the simulation studies. The I² test was significantly less specific than statistical significance testing for identifying the absence of selection bias. I² point estimates correlated inversely with p-values from statistical significance testing, and I² selection bias categories caused 26% of ‘true positive’ trials being over-classified by one severity category. For these reasons, negative test results may be helpful in ruling out baseline imbalances and selection bias in RCTs. However, due to its specificity below 80%, especially in terms of selection bias detection, as well as its tendency to overestimate true selection bias severity, positive I^2^ test results need to be considered with caution when appraising selection bias risk in RCTs during systematic reviews.

## Appendices

All data (Sections 1-6) are made fully available without restriction and can be freely downloaded from https://data.mendeley.com/datasets/gt28374pcf/1
